# Mapping-friendly sequence reductions: Going beyond homopolymer compression

**DOI:** 10.1016/j.isci.2022.105305

**Published:** 2022-10-13

**Authors:** Luc Blassel, Paul Medvedev, Rayan Chikhi

**Affiliations:** 1 Sequence Bioinformatics, Department of Computational Biology, Institut Pasteur, Paris, France; 2 Sorbonne Université, Collège doctoral, Paris F-75005, France; 3 Department of Computer Science and Engineering, Pennsylvania State University, University Park, PA, USA; 4 Department of Biochemistry and Molecular Biology, Pennsylvania State University, University Park, PA, USA; 5 Center for Computational Biology and Bioinformatics, Pennsylvania State University, University Park, PA, USA

**Keywords:** Biological sciences, Molecular biology, Biological sciences research methodologies, Transcriptomics

## Abstract

Sequencing errors continue to pose algorithmic challenges to methods working with sequencing data. One of the simplest and most prevalent techniques for ameliorating the detrimental effects of homopolymer expansion/contraction errors present in long reads is homopolymer compression. It collapses runs of repeated nucleotides, to remove some sequencing errors and improve mapping sensitivity. Though our intuitive understanding justifies why homopolymer compression works, it in no way implies that it is the best transformation that can be done. In this paper, we explore if there are transformations that can be applied in the same pre-processing manner as homopolymer compression that would achieve better alignment sensitivity. We introduce a more general framework than homopolymer compression, called mapping-friendly sequence reductions. We transform the reference and the reads using these reductions and then apply an alignment algorithm. We demonstrate that some mapping-friendly sequence reductions lead to improved mapping accuracy, outperforming homopolymer compression.

## Highlights

• Mapping-friendly sequence reductions (MSRs) are functions that transform DNA sequences
• They are a generalization of the concept of homopolymer compression
• We show that some well-chosen MSRs enable more accurate long-read mapping

## Graphical abstract

**Figure:**
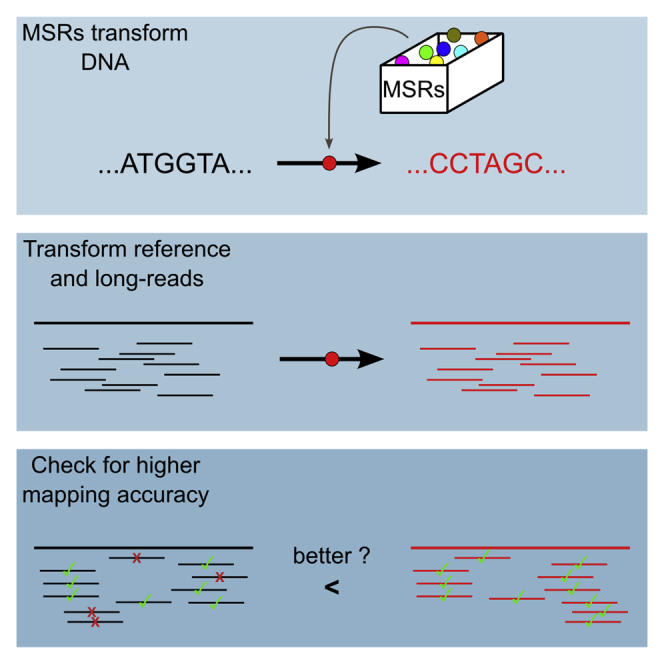

## Introduction

Sequencing errors continue to pose algorithmic challenges to methods working with read data. In short-read technologies, these tend to be substitution errors, but in long reads, these tend to be short insertions and deletions; most common are expansions or contractions of homopolymers (i.e. reporting 3 As instead of 4) (Dohm et al., 2020). Many algorithmic problems, such as alignment, become trivial if not for sequencing errors (Gusfield, 1997). Error correction can often decrease the error rate but does not eliminate all errors. Most tools therefore incorporate the uncertainty caused by errors into their underlying algorithms. The higher the error rate, the more detrimental its effect on algorithm speed, memory, and accuracy. While the sequencing error rate of any given technology tends to decrease over time, new technologies entering the market typically have high error rates (e.g. Oxford Nanopore Technologies). Finding better ways to cope with sequencing error therefore remains a top priority in bioinformatics.

One of the simplest and most prevalent techniques for ameliorating the detrimental effects of homopolymer expansion/contraction errors is *homopolymer compression* (HPC). HPC simply transforms runs of the same nucleotide within a sequence into a single occurrence of that nucleotide. For example, HPC applied to the sequence AAAGGTTA yields the sequence AGTA. To use HPC in an alignment algorithm, one first compresses the reads and the reference, then aligns each compressed read to the compressed reference, and finally reports all alignment locations, converted into the coordinate system of the uncompressed reference. HPC effectively removes homopolymer expansion/contraction errors from the downstream algorithm. Though there is a trade-off with specificity of the alignment (e.g. some of the compressed alignments may not correspond to true alignments) the improvement in mapping sensitivity usually outweighs it (Li, 2018).

The first use of HPC that we are aware of was in 2008 as a pre-processing step for 454 pyrosequencing data in the Celera assembler (Miller et al., 2008). It is used by a wide range of error-correction algorithms, e.g. for 454 data (Bragg et al., 2012), PacBio data (Au et al., 2012), and Oxford Nanopore data (Sahlin and Medvedev, 2021). HPC is used in alignment, e.g. by the widely used minimap2 aligner (Li, 2018). HPC is also used in long-read assembly, e.g. HiCanu (Nurk et al., 2020), SMARTdenovo (Liu et al., 2021), or mdBG (Ekim et al., 2021). HPC is also used for clustering transcriptome reads according to gene family of origin (Sahlin and Medvedev, 2020). Overall, HPC has been widely used, with demonstrated benefits.

Though our intuitive understanding justifies why HPC works, it in no way implies that it is the best transformation that can be done. Are there transformations that can be applied in the same pre-processing way as HPC that would achieve better alignment sensitivity? In this work, we define a more general notion which we call *mapping-friendly sequence reductions*. In order to efficiently explore the performance of all reductions, we identify two heuristics to reduce the search space of reductions. We then identify a number of mapping-friendly sequence reductions which are likely to yield better mapping performance than HPC. We evaluate them using two mappers (minimap2 and winnowmap2) on three simulated datasets (whole human genome, human centromere, and whole *Drosophila* genome). We show that some of these functions provide vastly superior performance in terms of correctly placing high mapping quality reads, compared to either HPC or using raw reads. For example, one function decreased the mapping error rate of minimap2 by an order of magnitude over the entire human genome, keeping an identical fraction of reads mapped.

We also evaluate whether HPC sensitivity gains continue to outweigh the specificity cost with the advent of telomere-to-telomere assemblies (Nurk et al., 2022). These contain many more low-complexity and/or repeated regions such as centromeres and telomeres. HPC may increase mapping ambiguity in these regions by removing small, distinguishing, differences between repeat instances. Indeed, we find that neither HPC nor our mapping-friendly sequence reductions perform better than mapping raw reads on centromeres, hinting at the importance of preserving all sequence information in repeated regions.

## Results

### Streaming sequence reductions

We wish to extend the notion of homopolymer compression to a more general function while maintaining its simplicity. What makes HPC simple is that it can be done in a streaming fashion over the sequence while maintaining only a local context. The algorithm can be viewed simply as scanning a string from left to right and, at each new character, outputting that character if and only if it is different from the previous character. In order to prepare for generalizing this algorithm, let us define a function $g^{\text{HPC}}:{\text{Σ}}^{2}\to \text{Σ}\cup \left\{\varepsilon \right\}$ where Σ is the DNA alphabet, ε is the empty character, and

$$g^{\text{HPC}}\left(x_{1}\cdot x_{2}\right)=\left\{ \begin{array}{ll} x_{2} & \text{if }x_{1}\neq x_{2}\\ \varepsilon & \text{if }x_{1}=x_{2} \end{array}\right..$$

Now, we can view HPC as sliding a window of size 2 over the sequence and at each new window, applying $g^{\text{HPC}}$ to the window and concatenating the output to the growing compressed string. Formally, let *x* be a string, which we index starting from 1. Then, the HPC transformation is defined as

(Equation 1) f(x)=x[1,ℓ−1]⋅g(x[1,ℓ])⋅g(x[2,ℓ+1])⋯g(x[|x|−ℓ+1,|x|])

where ℓ=2 and $g=g^{\text{HPC}}$. In other words, *f* is the concatenation of the first ℓ−1 characters of *x* and the sequence of outputs of *g* applied to a sliding window of length ℓ over *x*. The core of the transformation is given by *g* and the size of the context ℓ, and *f* is simply the wrapper for *g* so that the transformation can be applied to arbitrary length strings.

With this view in mind, we can generalize HPC while keeping its simplicity by 1) considering different functions *g* that can be plugged into Equations 1 and 2) increasing the context that *g* uses (i.e. setting ℓ>2). Formally, for a given alphabet Σ and a context size ℓ, a function *T* mapping strings to strings is said to be an *order-*ℓ *streaming sequence reduction* (*SSR*) if there exists some $g:{\text{Σ}}^{\ell }\to \text{Σ}\cup \left\{\varepsilon \right\}$ such that T=f.

**Figure 1:**
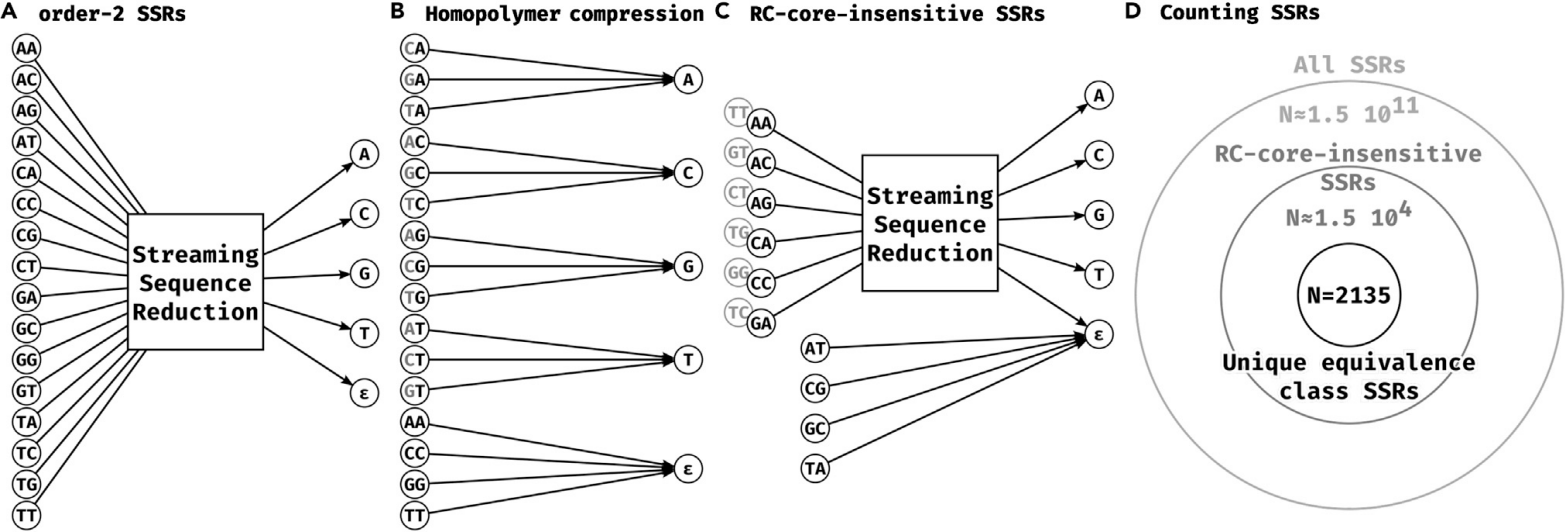
Representing and counting streaming sequence reductions (A) General representation of an order-2 streaming sequence reduction as a mapping of 16 input dinucleotides, to the 4 nucleotide outputs and the empty character ε. (B) Homopolymer compression is an order-2 SSR. All dinucleotides except those that contain the same nucleotide twice map to the second nucleotide of the pair. The 4 dinucleotides that are the two same nucleotides map to the empty character ε. (C) Our RC-core-insensitive order-2 SSRs are mappings of the 6 representative dinucleotide inputs to the 4 nucleotide outputs and the empty character ε. The 4 dinucleotides that are their own reverse complement are always mapped to ε. The remaining 6 dinucleotides are mapped to the complement of the mapped output of the reverse complement dinucleotide input. For example, if AA is mapped to C, then TT (the reverse complement of AA) will be mapped to G (the complement of C). (D) Number of possible SSR mappings under the different restrictions presented in the main text. All mappings from 16 dinucleotide inputs to 5 outputs (as in panel A) are represented by the outermost circle. All RC-core-insensitive mappings (as in panel C) are represented by the medium circle. All RC-core-insensitive mappings with only one representative of each equivalence class are represented by the innermost circle.

Figure 1A shows how an SSR can be visualized as a directed graph. Observe that an order-ℓ SSR is defined by a mapping between $|\text{Σ}{|}^{\ell }$ inputs and |Σ|+1 outputs. For example, for ℓ=2, there are n=16 inputs and k=5 outputs. Figure 1B visualizes HPC in this way

Since we aim to use SSRs in the context of sequencing data, we need to place additional restrictions on how they handle reverse complements. For example, given two strings *x* (e.g. a read) and *y* (e.g. a substring of the reference), a mapper might check if x=RC(y). When strings are pre-processed using an SSR *f*, it will end up checking if f(x)=RC(f(y)). However, x=RC(y) only implies that f(x)=f(RC(y)). In order to have it also imply that f(x)=RC(f(y)), we need *f* to be commutative with RC, i.e. applying SSR then RC needs to be equivalent to applying RC then SSR. We say that *f* is *RC insensitive* if for all *x*, f(RC(x))=RC(f(x)). Observe that HPC is RC insensitive.

### Restricting the space of streaming sequence reductions

To discover SSRs that improve mapping performance, our strategy is to put them all to the test by evaluating the results of an actual mapping software over a simulated test dataset reduced by each SSR. However, even with only 16 inputs and 5 outputs, the number of possible *g* mappings for order-2 SSRs is $5^{16}\approx 1.5\cdot 10^{11}$, which is prohibitive to enumerate. In this section, we describe two ideas for reducing the space of SSRs that we will test. In subsection reverse complement-core-insensitive streaming sequence reductions, we show how the restriction to RC-insensitive mappings can be used to reduce the search space. In subsection equivalence classes of SSRs, we exploit the natural symmetry that arises due to Watson-Crick complements to further restrict the search space.

These restrictions reduce the number of order-2 SSRs to only 2135, making it feasible to test all of them. Figure 1D shows an overview of our restriction process.

#### Reverse complement-core-insensitive streaming sequence reductions

Consider an SSR defined by a function *g*, as in Equation 1. Throughout this paper, we will consider SSRs that have a related but weaker property than RC-insensitive. We say that an SSR is *RC-core-insensitive* if the function *g* that defines it has the property that for every ℓ-mer *x* and its reverse complement *y*, we have that either g(x) is the reverse complement of g(y) or g(x)=g(y)=ε. We will restrict our SSR search space to RC-core-insensitive reductions in order to reduce the number of SSRs we will need to test.

Let us consider what this means for the case of ℓ=2, which will be the focal point of our experimental analysis. There are 16 ℓ-mers (i.e. dinucleotides) in total. Four of them are their own reverse complement: AT, TA, GC, and CG. The RC-core-insensitive restriction forces *g* to map each of these to ε, since a single nucleotide output cannot be its own reverse complement. This leaves 12 ℓ-mers, which can be broken down into 6 pairs of reverse complements. For each pair, we can order them in lexicographical order and write them as (AA,TT),(AC,GT),(AG,CT),(CA,TG),(CC,GG), and (GA,TC). Defining *g* can then be done by assigning an output nucleotide to the first ℓ-mer in each of these pairs and then assigning the complementary output nucleotide to the second ℓ-mer of each pair (Figure 1C). For example, we can define an SSR by assigning g(AA)=C, g(AC)=C, g(AG)=A, g(CA)=A, g(CC)=T, and g(GA)=G (implying that g(TT)=G, g(GT)=G, g(CT)=T, …). As an example, let us apply the corresponding SSR to an example read *r*:

$$\begin{array}{lllllll} r & = & \text{TAAGTTGA} & & f\left(RC\left(r\right)\right) & = & \text{TCACCTG}\\ f(r) & = & \text{TCAGGTG} & & RC\left(f\left(r\right)\right) & = & \;\text{CACCTGA}\\ \mathrm{RC}(r) & = & \text{TCAACTTA} & & & & \end{array}$$

Observe that the first ℓ−1 nucleotides of *r* (shown in red) are copied as-is, since we do not apply *g* on them (as per Equation 1). As we see in this example, this implies that f(RC(r)) is not necessarily equal to RC(f(r)); thus an RC-core-insensitive SSR is not necessarily an RC-insensitive SSR. However, an RC-core-insensitive SSR has the property that for all strings *r*, we have f(RC(r))[ℓ,|r|])=RC(f(r))[1,|r|−ℓ+1]. In other words, if we drop the ℓ−1 prefix of f(RC(r)) and the ℓ−1 suffix of RC(f(r)), then the two strings are equal. Though we no longer have the strict RC-insensitive property, this new property suffices for the purpose of mapping long reads. Since the length of the read sequences will be much greater than ℓ (in our results we will only use ℓ=2), having a mismatch in the first or last nucleotide will be practically inconsequential.

It is important to note though that there may be other RC-insensitive functions not generated by this construction. For instance, HPC cannot be derived using this method (as it does not map the dinucleotides AT, TA, GC, and CG to ε), and yet it is RC insensitive.

We can count the number of RC-core-insensitive SSR. Let us define i(ℓ) the number of input assignments necessary to fully determine the RC-core-insensitive SSR; one can think of this as the degrees-of-freedom in choosing *g*. As we showed, for ℓ=2, we have i(ℓ)=6. The number of RC-core-insensitive SSR is then $5^{i\left(\ell \right)}$. Therefore, for ℓ=2, instead of $5^{16}$ possible mappings, we have at most $5^{6}\approx 1.5\cdot 10^{4}$ RC-core-insensitive mappings (Figure 1D). For an odd ℓ>2, there are no ℓ-mers that are their own reverse complements, hence $i\left(\ell \right)=4^{\ell }/2$. If ℓ is even, then there are $4^{\ell /2}$ inputs that are their own reverse complements (i.e. we take all possible sequences of length ℓ/2 and reconstruct the other half with reverse complements). Thus, $i\left(\ell \right)=\left(4^{\ell }-4^{\ell /2}\right)/2$.

#### Equivalence classes of SSRs

Non-mapping-related preliminary tests led us to hypothesize that swapping A↔T and/or C↔G, as well as swapping the whole A/T pair with the C/G pair in the SSR outputs would have a negligible effect on performance. In other words, we could exchange the letters of the output in a way that preserves the Watson-Crick complementary relation. Intuitively, this can be due to the symmetry induced by reverse complements in nucleic acid strands, though we do not have a more rigorous explanation for this effect. In this section, we will formalize this observation by defining the notion of SSR equivalence. This will reduce the space of SSRs that we will need to consider by allowing us to evaluate only one SSR from each equivalence class.

Consider an RC-core-insensitive SSR defined by a function *g*, as in Equation 1. An ℓ-mer is canonical if it is not lexicographically larger than its reverse complement. Let *I* be the set of all ℓ-mers that are canonical. Such an SSR’s *dimension k* is the number of distinct nucleotides that can be output by *g* on inputs from *I* (not counting ε). The dimension can range from 1 to 4. Next, observe that *g* maps all elements of *I* to one of k+1 values (i.e. Σ∪ε). The output of *g* on ℓ-mers not in *I* is determined by its output on ℓ-mers in *I*, since we assume the SSR is RC-core-insensitive. We can therefore view it as a partition of *I* into k+1 sets $S_{0}$, …, $S_{k}$, and then having a function *t* that is an injection from {1,…,k} to Σ that assigns an output letter to each partition. Furthermore, we permanently assign the output letter for $S_{0}$ to be ε. Note that while $S_{0}$ could be empty, $S_{1},\ldots ,S_{k}$ cannot be empty by definition of dimension. For example, the SSR used in Section reverse complement-core-insensitive streaming sequence reductions has dimension four and corresponds to the partition $S_{0}=\left\{\right\},S_{1}=\left\{AG\text{,}CA\right\}$, $S_{2}=\left\{CC\right\}$, $S_{3}=\left\{AA\text{,}AC\right\}$, and $S_{4}=\left\{GA\right\}$, and to the injection t(1)=A, t(2)=T, t(3)=C, and t(4)=G.

Let ISCOMP(x,y) be a function that returns true if two nucleotides x,y∈Σ∪{ε} are Watson-Crick complements, and false otherwise. Consider two SSRs of dimension *k* defined by $S_{0},\ldots ,S_{k},t$ and $S_{0}^{\text{′}},\ldots ,S_{k}^{\text{′}},t^{\text{′}}$, respectively. We say that they are equivalent if all the following conditions are met:

• $S_{0}=S_{0}^{\text{′}}$,
• there exists a permutation π of {1,…,k} such that for all 1≤i≤k, we have $S_{i}=S_{\pi \left(i\right)}^{\text{′}}$,
• for all 1≤i<j≤k, we have $\text{ISCOMP}\left(t\left(i\right)\text{,}t\left(j\right)\right)=\text{ISCOMP}\left(t^{\text{′}}\left(\pi \left(i\right)\right)\text{,}t^{\text{′}}\left(\pi \left(j\right)\right)\right)$.

One can verify that this definition is indeed an equivalence relation, i.e. it is reflexive, symmetric, and transitive. Therefore, we can partition the set of all SSRs into equivalence classes based on this equivalence relation. One caveat is that a single SSR defined by a function *g* may correspond to multiple SSRs of the form $S_{0},\ldots ,S_{k},t$. However, these multiple SSRs are equivalent; hence, the resulting equivalence classes are not affected. Furthermore, we can assume that there is some rule to pick one representative SSR for its equivalence class; the rule itself does not matter in our case.

**Figure 2:**
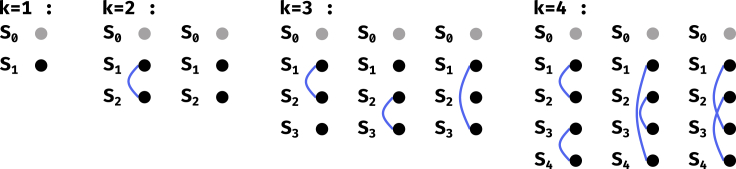
SSR equivalence classes for a fixed partition of the inputs $S_{0}$ is always assigned ε, so it is represented by a gray node. A blue link between $S_{i}$ and an $S_{j}$ denotes that ISCOMP(t(i),t(j))=true. The equivalence classes are determined by the Watson-Crick complementary relationships between the rest of the parts, i.e. by all the possible ways to draw the blue links.

Figure 2 shows the equivalence classes for ℓ=2, for a fixed partition. An equivalence class can be defined by which pair of classes $S_{i}$ and $S_{j}$ have complementary outputs under *t* and $t^{\text{′}}$. Let us define o(k) as the number of equivalence classes for a given partition and a given *k*. Then Figure 2 shows that o(1)=1, o(2)=2, and o(3)=o(4)=3. There are thus only 9 equivalence classes for a given partition.

#### Counting the number of restricted SSRs

In this section, we derive a formula for the number of restricted SSRs, i.e. SSRs that are RC-core-insensitive and that are representative for their equivalence class. Consider the class of RC-core-insensitive SSRs with dimension *k*. In subsection reverse complement-core-insensitive streaming sequence reductions, we derived that the degrees-of-freedom in assigning ℓ-mers to an output is $i\left(\ell \right)=4^{\ell }/2$ if ℓ is odd and $i\left(\ell \right)=\left(4^{\ell }-4^{\ell /2}\right)/2$ if ℓ is even. Let C(ℓ,k) be the number of ways that i(ℓ) ℓ-mers can be partitioned into k+1 sets $S_{0},\ldots ,S_{k}$, with $S_{1},\ldots ,S_{k}$ required to be non-empty. Then, in subsection equivalence classes of SSRs, we have derived o(k), the number of SSR equivalence classes for each such partition. The number of restricted SSRs can then be written as

(Equation 2) $$N\left(\ell \right)=\sum_{k=1}^{4}C\left(\ell \text{,}k\right)\cdot o\left(k\right)$$

To derive the formula for C(ℓ,k), we first recall that the number of ways to partition *n* elements into *k* non-empty sets is known as the Stirling number of the second kind and is denoted by $\left\{\begin{array}{l} n\\ k \end{array}\right\}$ (Graham et al., 1994, p.265). It can be computed using the formula

$$\left\{\begin{array}{l} n\\ k \end{array}\right\}=\frac{1}{k!}\overset{k}{\underset{i=0}{\sum }}{\left(-1\right)}^{i}\left(\begin{array}{l} k\\ i \end{array}\right){\left(k-i\right)}^{n}$$

Let *j* be the number of the i(ℓ) ℓ-mers that are assigned to $S_{0}$. Note, this does not include the ℓ-mers that are self-complementary that are forced to be in $S_{0}$. Let C(ℓ,k,j) be the number of ways that i(ℓ) ℓ-mers can be partitioned into k+1 sets $S_{0},\ldots ,S_{k}$, such that *j* of the ℓ-mers go into $\left|S_{0}\right|$ and $S_{1},\ldots ,S_{k}$ to are non-empty. We need to consider several cases depending on the value of *j*:

• In the case that j=0, we are partitioning the i(ℓ) inputs among non-empty sets $S_{1},\ldots ,S_{k}$. Then $C\left(\ell \text{,}k\text{,}j\right)=\left\{\begin{array}{l} i\left(\ell \right)\\ k \end{array}\right\}$.
• In the case that 1≤j≤i(ℓ)−k, there are $\left(\begin{array}{l} i\left(\ell \right)\\ j \end{array}\right)$ ways to choose which *j* ℓ-mers are in $S_{0}$, and $\left\{\begin{array}{l} i\left(\ell \right)-j\\ k \end{array}\right\}$ ways to partition the remaining ℓ-mers into $S_{1},\ldots ,S_{k}$. Hence, $C\left(\ell \text{,}k\text{,}j\right)=\left(\begin{array}{l} i\left(\ell \right)\\ j \end{array}\right)\left\{\begin{array}{l} i\left(\ell \right)-j\\ k \end{array}\right\}$.
• In the case that j>i(ℓ)−k, it is impossible to partition the remaining *k* (or fewer) ℓ-mers into $S_{1},\ldots ,S_{k}$ such that the sets are non-empty. Recall that as we assume the dimension is *k*, each set must contain at least one element. Hence, C(ℓ,k,j)=0.

Putting this together into Equation 2, we get

$$N\left(\ell \right)=\overset{4}{\underset{k=1}{\sum }}o\left(k\right)\left(\left\{\begin{array}{l} i\left(\ell \right)\\ k \end{array}\right\}+\overset{i\left(\ell \right)-k}{\underset{j=1}{\sum }}\left(\begin{array}{l} i\left(\ell \right)\\ j \end{array}\right)\left\{\begin{array}{l} i\left(\ell \right)-j\\ k \end{array}\right\}\right)$$

For ℓ=2, we have N(2)=2135 restricted SSRs, which is several orders of magnitude smaller than the initial $5^{16}$ possible SSRs and allows us to test the performance of all of them. For order-3 SSRs, we get $N\left(3\right)=2.9\cdot 10^{21}$ which is much smaller than the full search space of $5^{4^{3}}\approx 5.4\cdot 10^{44}$; for order-4 SSRs, we get a similar reduction in search space with $N\left(4\right)=9.4\cdot 10^{84}$ as opposed to the full search space of $5^{4^{4}}\approx 8.6\cdot 10^{178}$. For these higher order SSRs, although the restricted search space is much smaller than the full original one, it is still too large to exhaustively search.

### Selection of mapping-friendly sequence reductions

We selected a set of “promising” SSRs starting from all of the 2135 SSRs enumerated in Section restricting the space of streaming sequence reductions, that we call *mapping-friendly sequence reductions* (*MSR*). The selection was performed on a 0.5× coverage read set, simulated from a whole human genome assembly (Nurk et al., 2022). The transformed reads were mapped to the transformed reference using minimap2 and paftools mapeval (Li, 2018) was used to compute a mapping error rate. Note that overfitting SSRs to a particular genome is acceptable in applications where a custom SSR can be used for each genome. Yet in this work, the same set of selected SSR will be used across all genomes.

**Figure 3:**
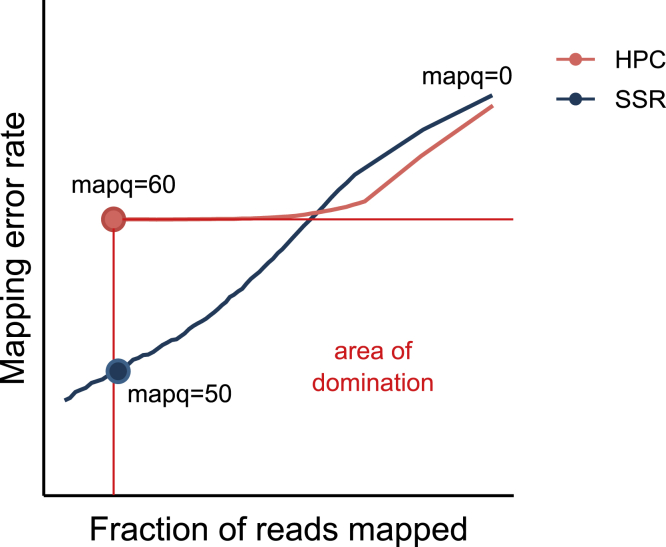
Illustration of how a respective mapq threshold is chosen for each of our evaluated SSRs The orange dot labeled ”mapq = 60″ shows the mapping error rate and fraction of reads mapped for HPC at mapq threshold 60. Anything below and to the right of this point is strictly better than HPC at mapq = 60, i.e. it has both a lower mapping error rate and higher fraction of reads mapped. If an evaluated SSR does not pass through this region, then it is discarded from further consideration. In the figure, the blue SSR does pass through this region, indicating that it is better than HPC at mapq = 60. We identify the leftmost point (marked as a blue dot) and use the mapq threshold at that point as the respective threshold.

For each evaluated SSR, we selected, if it exists, the highest mapq threshold for which the mapped read fraction is higher and the mapping error rate is lower than HPC at mapq 60 (0.93 and $2.1\cdot 10^{-3}$, respectively), Figure 3 illustrates the idea. Then, we identified the 20 SSRs that have the highest fraction of reads mapped at their respective thresholds. Similarly, we identified the 20 SSRs with the lowest mapping error rate. Finally, we select the 20 SSRs that have the highest percentage of thresholds “better” than HPC at mapq 60; i.e. the number of mapq thresholds for which the SSR has both a higher fraction of reads mapped and lower mapping error rate than HPC at a mapq threshold of 60, divided by the total number of thresholds (=60).

The union of these 3 sets of 20 SSRs resulted in a set of 58 “promising” MSRs. Furthermore, we will highlight three MSRs that are “best in their category”, i.e.

• **MSR**_**F**_**:** The MSR with the highest fraction of mapped reads at a mapq threshold of 0.
• **MSR**_**E**_**:** The MSR with the lowest mapping error rate at its respective mapq threshold.
• **MSR**_**P**_**:** The MSR with the highest percentage of mapq thresholds for which it is “better” than HPC at mapq 60.

**Figure 4:**
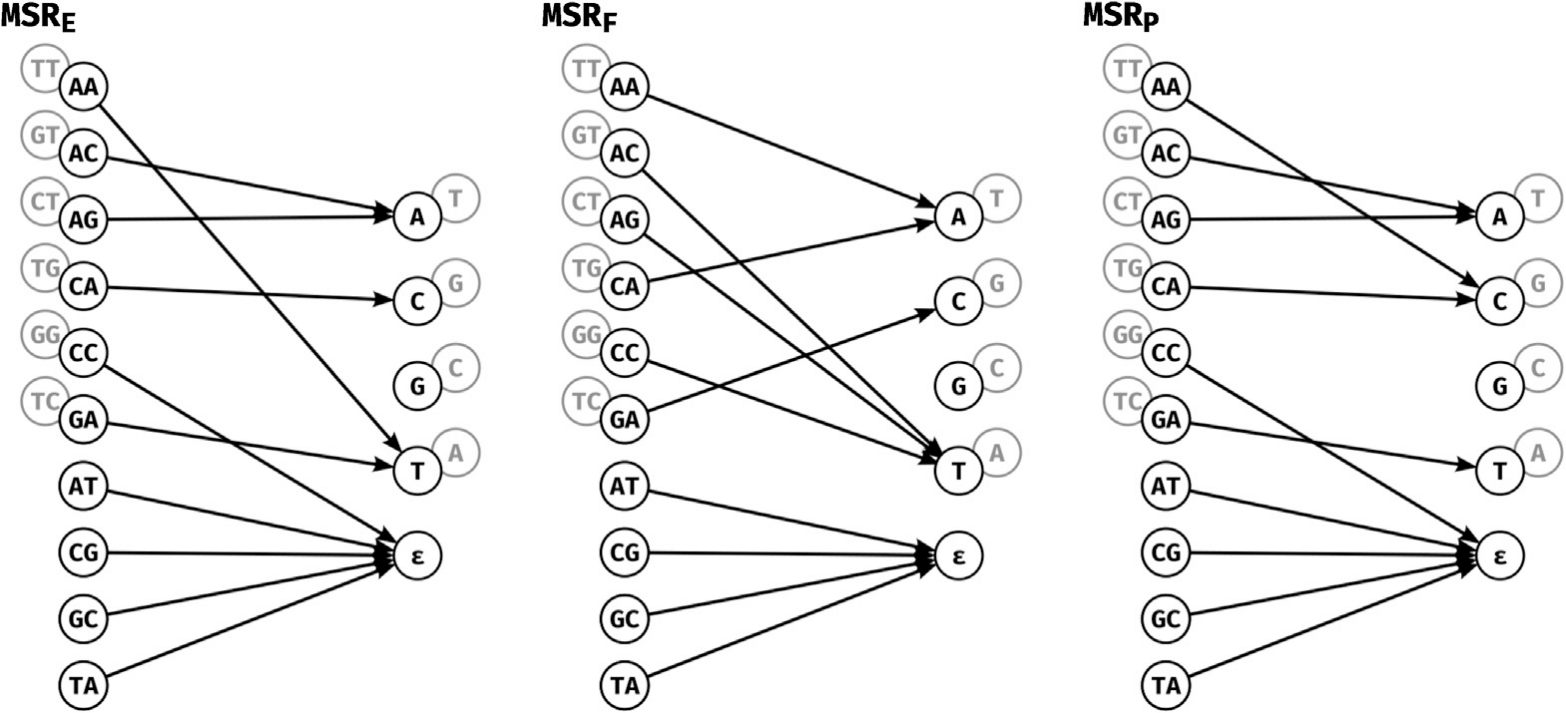
Graph representations of our highlighted MSRs: MSR_E_**, MSR**_F_**, and MSR**_P_ MSR_E_ has the lowest mapping error rate of among MSRs at the highest mapq threshold for which it performs better than HPC at mapq 60, MSR_F_ has the highest fraction of reads mapped at mapq 60 and MSR_P_ has the highest percentage of mapq thresholds for which it outperforms HPC at mapq 60. The grayed out nodes represent the reverse complement of input dinucleotides and outputs, as in Figure 1C. For example for MSR_E_, AA is mapped to T, so TT is mapped to A.

Figure 4 shows the actual functions MSR_F_, MSR_E_, and MSR_P_. An intriguing property is that they output predominantly As and Ts, with MSR_P_ assigning only 2 input pairs to the G/C output whereas MSR_E_ and MSR_F_ assign only one. Additionally, MSR_E_ and MSR_P_ both assign the {CC,GG} input pair to the deletion output ε removing any information corresponding to repetitions of either G or C from the reduced sequence. Overall, this means the reduced sequences are much more AT-rich than their raw counterparts, but somehow information pertinent to mapping is retained.

The 58 selected MSRs, as well as HPC and the identity transformation (denoted as *raw*), were then evaluated on larger read-sets simulated from 3 whole genome references: a whole human genome assembly (Nurk et al., 2022), a whole *Drosophila melanogaster* genome assembly (Adams et al., 2000), and a synthetic centromeric sequence (Mikheenko et al., 2020) (see STAR methods for more details).

### Mapping-friendly sequence reductions lead to lower mapping errors on whole genomes

**Figure 5:**
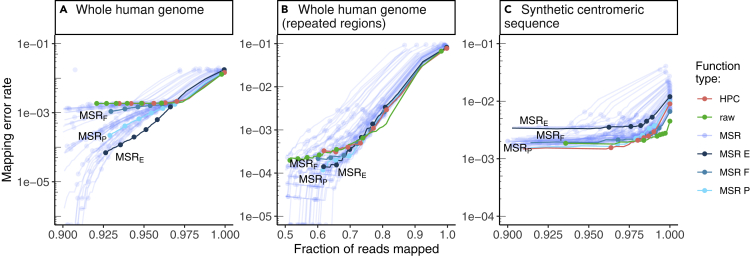
Performance of our 58 selected mapping-friendly sequence reductions across genomes on reads simulated by nanosim (Panel A) shows the whole human genome assembly, (B and C) the subset of mapped reads from panel B that originate from repetitive regions, and C) the “TandemTools” synthetic centromeric reference sequence. We highlighted the best-performing mapping-friendly sequence reductions as MSR E, F, and P, respectively, in terms of cumulative mapeval mapping error rate, fraction of reads mapped, and percentage of better thresholds than HPC. Each point on a line represents, from left to right, the mapping quality thresholds 60, 50, 40, 30, 20, 10, and 0. For the first point of each line, only reads of mapping quality 60 are considered, and the y value represents the rate of these reads that are not correctly mapped, the x value represents the fraction of reads that are mapped at this threshold. The next point is computed for all reads of mapping quality ≥50, etc. The rightmost point on any curve represents the mapping error rate and the fraction of mapped reads for all primary alignments. The x-axes are clipped for lower mapped read fractions to better differentiate HPC, raw and MSRs E, F, and P. See Also Figure S7.

Across the entire human genome, at high mapping quality thresholds (above 50), our selected 58 MSRs generally have lower mapping error rate than HPC and raw (Figure 5A and Table 1). This is not surprising, as we selected those MSRs partly on the criteria of outperforming HPC at mapq 60; however, it does demonstrate that we did not overfit to the simulated reads used to select the MSRs.

**Table 1:** Performance of MSRs, HPC, and raw mappings across different mappers and reference sequences

mapq		Whole human genome				Whole human genome				Whole Drosophila genome			
		minimap2				winnowmap2				minimap2			
		Fraction		error		fraction		error		fraction		error	
HPC	60	0.935	+0%	1.85e-03	+0%	0.894	+0%	1.43e-03	+0%	0.957	+0%	2.27e-03	+0%
raw	60	0.921	−1%	1.86e-03	+0%	**0.932**	+**4%**	1.75e-03	+23%	0.958	+0%	2.27e-03	−0%
MSR_F_	50	**0.938**	+**0%**	1.29e-03	−30%	0.886	−1%	3.82e-04	−73%	**0.960**	**+0%**	1.37e-03	−39%
MSR_E_	50	0.936	+0%	**1.17e-04**	−**94%**	0.820	−8%	**8.93e-05**	−**94%**	0.954	−0%	**0**	−**100%**
MSR_P_	50	**0.938**	**+0%**	4.15e-04	−78%	0.845	−6%	1.14e-04	−92%	0.957	+0%	8.11e-04	−64%

For each reference sequence and mapper pair, we report the fraction of reads mapped (“fraction” columns) and the mapping error rate (“error” columns) reported by paftools mapeval. The percentage differences are computed w.r.t the respective HPC value. The second column indicates that for HPC and raw these metrics were obtained for alignments of mapping quality of 60; for MSRs E, F, and P, these metrics were obtained for alignments of mapping quality ≥50. See also Table S1.

Mapping quality is only an indication from the aligner to estimate whether a read mapping is correct, and according to Figure 5A, the mapping error rate of most MSRs is low even for mapping qualities lower than 60. Therefore, we choose to compare MSR-mapped reads with lower mapping qualities against raw or HPC-mapped reads with the highest (60) mapping quality (which is the mapping quality thresholds most practitioners would use by default).

Table 1 shows that the three selected MSRs outperform both HPC and raw in terms of mapping error rate, with similar fractions of mapped reads overall. For example, on the human genome, at mapq ≥ 50, MSR_F_, MSR_P_, and MSR_E_ all map more reads than either HPC or raw at mapq = 60, and MSR_P_ and MSR_E_ also have mapping error rates an order of magnitude lower than either HPC or raw.

To evaluate the robustness of MSRs E, F, and P, we investigated the impact of mapping to a different organism or using another mapper. To this effect, we repeated the evaluation pipeline in these different settings:

• Using the *Drosophila melanogaster* whole genome assembly as reference and mapping with minimap2.
• Using the whole human genome assembly as reference but mapping with winnowmap2 (version 2.02) (Jain et al., 2020). The same options as minimap2 were used, and k-mers were counted using meryl (Rhie et al., 2020), considering the top 0.02% as repetitive (as suggested by the winnowmap2 usage guide).

MSRs E, F, and P behave very similarly in both of these contexts compared to HPC/raw: by selecting mapped reads with mapq ≥ 50 for the three MSRs, we obtain a similar fraction of mapped reads with much lower mapping error rates (Table 1). A noticeable exception is the winnowmap2 experiment, where a larger fraction of raw reads are mapped than any other MSR and even HPC. A more detailed result table can be found in Table S1, and a graph of MSR performance on the whole Drosophila genome in Figure S7. As Figure S7 shows, we also evaluated these MSRs on a whole *Escherichia coli* (GenBank: U00096.2 (Blattner et al., 1997)) genome, where we observed similar results, albeit the best MSRs do not seem to be one of our three candidates. This might mean that specific MSRs are more suited to particular types of genomes.

### Mapping-friendly sequence reductions increase mapping quality on repeated regions of the human genome

To evaluate the performance of our MSRs specifically on repeats, we extracted the reads for which the generating region overlapped with the repeated region of the whole human genome by more than 50% of the read length. We then evaluated the MSRs on these reads only. Repeated regions were obtained from https://t2t.gi.ucsc.edu/chm13/hub/t2t-chm13-v1.1/rmsk/rmsk.bigBed.

We obtained similar results as on the whole human genome, with MSRs E, F, and P performing better than HPC at mapq 50 (Figure 5B). At a mapq threshold of 50, the mapping error rate is 53%, 31%, and 39% lower than HPC at mapq 60 for MSRs E, F, and P, respectively, while the fraction of mapped reads remains slightly higher. At mapq = 60, raw has a mapping error rate 40% lower than HPC but the mapped fraction is also 17% lower.

### Raw mapping improves upon HPC on centromeric regions

On the “TandemTools” centromeric reference, HPC consistently maps a smaller fraction of reads than raw, across all mapping quality thresholds (Figure 5C). Additionally, the mapping error rate for raw is often inferior to that of HPC. The same is true for our selected MSRs: most of them have comparable performance to HPC, but none of them outperform raw mapping (Figure 5C).

We conjecture this is due to the highly repetitive nature of centromeres. HPC likely removes small unique repetitions in the reads and the reference that might allow mappers to better match reads to a particular occurrence in a centromeric pattern. Mapping raw reads on the other hand preserves all bases in the read and better differentiates repeats. Therefore, it seems inadvisable to use HPC when mapping reads to highly repetitive regions of a genome, such as a centromere.

### Positions of incorrectly mapped reads across the entire human genome

**Figure 6:**
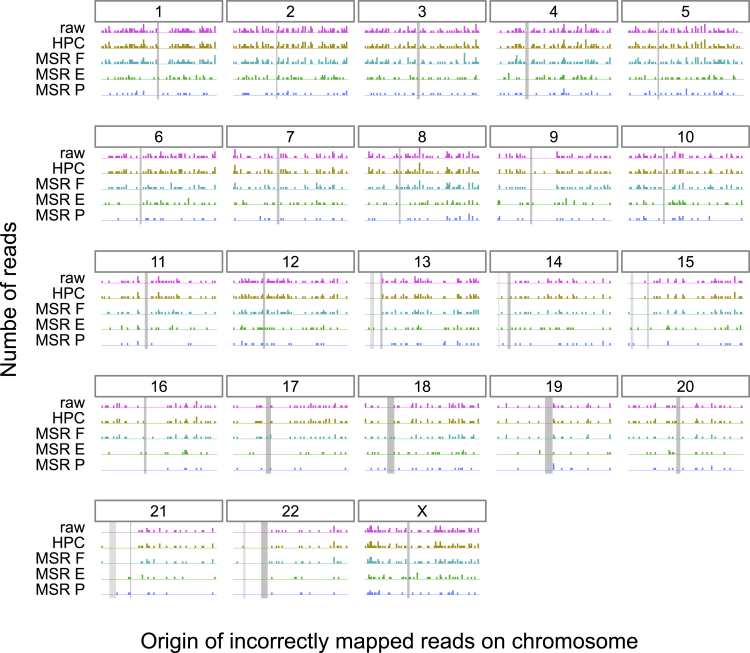
Histogram of the original simulated positions for the incorrectly mapped reads using minimap2 at high mapping qualities across the whole human genome, for several transformation methods For a given chromosome, each row represents the number of simulated reads starting at that particular region. The dark gray rectangle represents the position of the centromere for that chromosome, obtained from annotations provided by the T2T consortium (http://t2t.gi.ucsc.edu/chm13/hub/t2t-chm13-v1.1/). Similarly, for chromosomes 13, 14, 15, 21, and 22, a lighter gray rectangle represents the position of the “stalk” satellites also containing repetitive regions. For HPC and raw reads only alignments of mapping quality 60 were considered. To provide a fair comparison, alignments with mapping qualities ≥50 were considered for MSRs E, F, and P. See also Figures S1–S5.

To study how MSRs E, F, and P improve over HPC and raw mapping in terms of mapping error rate on the human genome, we selected all the primary alignments that paftools mapeval reported as incorrectly mapped. For HPC and raw, only alignments of mapping quality equal to 60 were considered. To report a comparable fraction of aligned reads, we selected alignments of mapping quality ≥50 for MSRs. We then reported the origin of those incorrectly mapped reads on whole human genome reference, shown per-chromosome in Figure 6.

We observe that erroneously mapped reads are not only those from centromeres but also originate from many other genomic regions. MSRs E and P have a markedly lower number of these incorrect mappings than either HPC or raw, with 1118 incorrect mappings for raw mappings and 1130 for HPC as opposed to 549, 970, and 361 for MSRs E, F, and P, respectively. This stays true even for difficult regions of the genome such as chromosome X, where raw and HPC have 70 incorrect mappings as opposed MSRs E and P that have 39 and 27 errors, respectively.

We also investigated where all simulated reads were mapped on the whole human genome assembly, for raw, HPC, and MSRs E,F, and P in Figures S2–S6. The correctly mapped reads are, as expected, evenly distributed along each chromosome. The incorrectly mapped reads are however unevenly distributed. For most chromosomes, there is a sharp peak in the distribution of incorrectly mapped reads, located at the position of the centromere. For the acrocentric chromosomes, there is a second peak corresponding to the “stalk” satellite region, with an enrichment of incorrectly mapped reads. This is expected since both centromeres and “stalks” are repetitive regions which are a challenge for mapping. For chromosomes 1, 9, and 16, however the majority of incorrectly mapped reads originate in repeated regions just after the centromere.

## Discussion

We have introduced the concept of mapping-friendly sequence reduction and shown that it improves the accuracy of the popular mapping tool minimap2 on simulated Oxford Nanopore long reads.

We focused on reads with high mapping quality (50–60), as it is a common practice to disregard reads with low mapping quality (Prodanov and Bansal, 2020; Li, 2021; Li et al., 2018). However, across all mapped reads (mapq ≥0), HPC and our MSRs have lower mapping accuracies than raw reads, consistent with the recommendation made in minimap2 to not apply HPC to ONT data. Despite this, we newly show the benefit of using HPC (and our MSRs) with minimap2 on ONT data when focusing on high mapping quality reads. Furthermore, MSRs provide a higher fraction of high-mapq reads compared to both raw and HPC, as shown on the human and Drosophila genomes.

A natural future direction is to also test whether our MSRs perform well on mapping Pacific Biosciences long reads. Furthermore, it is important to highlight that our sampling of MSRs is incomplete. This is of course due to only looking at functions having l=2, but also to the operational definition of RC-core-insensitive functions, and finally to taking representatives of equivalence classes. An interesting future direction would consist in exploring other families of MSRs, especially those that would include HPC and/or close variations of it.

Additionally, our analyses suggests to not perform HPC on centromeres and other repeated regions, hinting at applying sequence transformations to only some parts of the genomes. We leave this direction for future work.

### Limitations of the study

Our proposed MSRs improve upon HPC at mapq 60, both in terms of fraction of reads mapped and mapping error rate on whole human, *Drosophila melanogaster*, and *Escherichia coli* genomes. We chose these sequences because they were from organisms that we deemed different enough; however, it would be interesting to verify if our proposed MSRs are still advantageous on even more organisms, e.g. more bacterial or viral genomes. This would allow us to assess the generalizability of our proposed MSRs.

We made the choice of using simulated data to be able to compute a mapping error rate. Some metrics, such as fraction of reads mapped, might still be informative with regards to the mapping performance benefits of MSRs, even on real data. Evaluating the MSRs on real data might be more challenging but would offer insight into real-world usage of such pre-processing transformations.

The hypothesis we made in subsection equivalence classes of SSRs was derived from non-mapping-related tests; it helped us reduce the search space and find MSRs. Testing if this hypothesis holds true on mapping tasks would help us make sure we are not missing some potentially well-performing SSRs by discarding them at this stage.

Finally, the restrictions we imposed to define RC-core-insensitive MSRs though intuitively understandable are somewhat arbitrary, so exploring a larger search space might be beneficial. Alternatively, for higher order MSRs, even with our restrictions, the search spaces remain much too large to be explored exhaustively. To mitigate this problem, either further restrictions need to be found, or an alternative, optimization-based exploration method should be implemented.

## STAR★Methods

### Key resources table

**Table:** 

REAGENT or RESOURCE	SOURCE	IDENTIFIER
**Deposited data**		

T2T CHM13 v1.1, whole human genome assembly	(Nurk et al., 2022)	GenBank: GCA_009914755.3
Release 6 plus ISO1 MT, whole drosophila melanogaster genome assembly	(Adams et al., 2000)	GenBank: GCA_000001215.4
Synthetic centrormeric sequence	(Mikheenko et al., 2020)	https://github.com/ablab/TandemTools/blob/master/test_data/simulated_del.fasta
*Escherichia coli* str. K-12 substr. MG1655, complete genome	(Blattner et al., 1997)	GenBank: U00096.2
Coordinates of repeated regions of the CHM13 whole genome assembly	Telomere to Telomere consortium	https://t2t.gi.ucsc.edu/chm13/hub/t2t-chm13-v1.1/rmsk/rmsk.bigBed

**Software and algorithms**		

minimap2 v2.22-r1101	(Li, 2018)	https://github.com/lh3/minimap2
Winnowmap v2.0	(Jain et al., 2020)	https://github.com/marbl/Winnowmap
NanoSim v3.0.0	(Yang et al., 2017)	https://github.com/bcgsc/NanoSim
Bedtools v2.30.0	(Quinlan and Hall et al., 2010)	https://github.com/arq5x/bedtools2
Meryl v1.0	(Rhie et al., 2020)	https://github.com/marbl/Winnowmap
Analysis pipelines	This paper	https://doi.org/10.5281/zenodo.6859636

### Resource availability

#### Lead contact

Further information and requests for resources should be directed to and will be fulfilled by the lead contact, Rayan Chikhi (rayan.chikhi@pasteur.fr)

#### Materials availability

This study did not generate new unique reagents.

### Method details

#### Datasets

The following three reference sequences were used for evaluation:

##### Whole human genome

This reference sequence is a whole genome assembly of the CHM13hTERT human cell line by the Telomere-to-Telomere consortium (Nurk et al., 2022). We used the 1.1 assembly release (Genbank Assembly ID: GCA_009914755.3).

##### Whole *Drosophila* genome

This reference sequence is a whole genome assembly of a *Drosophila melanogaster*, release 6.35 (Genbank Assembly ID: GCA_000001215.4) (Adams et al., 2000).

##### Synthetic centromeric sequence

This sequence was obtained from the TandemTools mapper test data (Mikheenko et al., 2020). It is a simulated centromeric sequence that is inherently difficult to map reads to. Document S1 describes how it was constructed, and it is downloadable from https://github.com/lucblassel/TandemTools/blob/master/test_data/simulated_del.fasta.

#### Simulation pipeline

Given a reference sequence, simulated reads were obtained using nanosim (Yang et al., 2017) with the human_NA12878_DNA_FAB49712_guppy_flipflop pre-trained model, mimicking sequencing with an Oxford Nanopore instrument. The number of simulated reads was chosen to obtain a theoretical coverage of whole genomes around 1.5×, this resulted in simulating $\approx 6.6\cdot 10^{5}$ reads for the whole human genome and $\approx 2.6\cdot 10^{4}$ reads for the whole Drosophila genome. Since the centromeric sequence is very short, we aimed for a theoretical coverage of 100× which resulted in $\approx 1.3\cdot 10^{4}$ simulated reads.

For each evaluated SSR, the reads as well as the reference sequence were reduced by applying the SSR to them. The reduced reads were then mapped to the reduced reference using minimap2 (Li, 2018) with the map-ont preset and the -c flag to generate precise alignments. Although HPC is an option in minimap2 we do not use it and we evaluate HPC as any of the other SSRs by transforming the reference and reads prior to mapping. The starting coordinates of the reduced reads on the reduced reference were translated to reflect deletions incurred by the reduction process. The mapping results with translated coordinates were filtered to keep only the primary alignments. This process was done for each of our 2135 SSRs as well as with HPC and the original untransformed reads (denoted as *raw*).

#### Evaluation pipeline

We use two metrics to evaluate the quality of a mapping of a simulated read set. The first is the *fraction of reads mapped*, i.e. that have at least one alignment. The second is the *mapping error rate*, which is the fraction of mapped reads that have an incorrect location as determined by paftools mapeval (Li, 2018). This tool considers a read as correctly mapped if the intersection between its true interval of origin, and the interval where it has been mapped to, is at least 10% of the union of both intervals.

Furthermore, we measure the mapping error rate as a function of a given *mapping quality threshold*. Mapping quality (abbreviated mapq) is a metric reported by the aligner that indicates its confidence in read placement; the highest value (60) indicates that the mapping location is likely correct and unique with high probability, and a low value (e.g. 0) indicates that the read has multiple equally likely candidate mappings and that the reported location cannot be trusted. The mapping error rate at a mapq threshold *t* is then defined as the mapping error rate of reads whose mapping quality is *t* or above. For example, the mapping error rate at t=0 is the mapping error rate of the whole read set, while the mapping error rate at t=60 is the mapping error rate of only the most confident read mappings. Observe that the mapping error rate decreases as *t* increases.

All experiments performed for this article are implemented and documented as nextflow workflows available in this project’s repository (github.com/lucblassel/MSR_discovery). These workflows may be used to rerun experiments and reproduce results. The repository also contains a Rmarkdown notebook to generate all figures and tables in the main text and supplemental information from the pipeline outputs.

## Data Availability

This paper analyzes existing, publicly available data. These accession numbers for the datasets are listed in the key resources table. All original code has been deposited at a github backed zenodo repository and is publicly available as of the date of publication. DOIs are listed in the key resources table, and the backing github repository is available at github.com/lucblassel/MSR_discovery. Any additional information required to reanalyze the data reported in this paper is available from the lead contact upon request.
