# Supplementary material for: Mapping-friendly sequence reductions: Going beyond homopolymer compression
Source: iScience. 2022 Oct 13;25(11):105305. doi: 10.1016/j.isci.2022.105305 (PMC9633736; doi:10.1016/j.isci.2022.105305)
Supplement: Document S1. Figures S1–S6 and Table S1 [file mmc1.pdf]

iScience, Volume *25*

Supplemental information

Mapping-friendly sequence reductions: Going beyond homopolymer compression

Luc Blassel, Paul Medvedev, and Rayan Chikhi

Document S1. “TandemTools” dataset generation, related to STAR Methods

This dataset was obtained by taking a human X chromosome HOR sequence, concatenating it 500 times with added mutations in order to obtain an approximately 1 Mbp long sequence. Then 1200 reads were simulated from the sequence using `nanosim` (Yang et al., 2017) and assembled using a centromere-tailored pipeline (Bzikadze and Pevzner, 2020). A 10kbp deletion was then added to this assembly. The resulting sequence is the one we refer to as the “Centromeric sequence”.

Table S1. MSR performance comparison, related to Table 1

Comparing performance of MSRs on the whole human genome, whole *Drosophila melanogaster* genome, repeated regions of the whole human genome and synthetic centromeric sequence. Results using `minimap2` (Li, 2018) and `winnowmap2` (Jain et al., 2020). The number of simulated reads for each reference sequence is given in parentheses and called n. Results are reported for mapq thresholds of 60, 50 and 0. The best performance for each category is highlighted in bold. The percentage difference are computed w.r.t HPC at each given threshold.

	mapq=60		mapq≥ 50		any mapq	
mapping friendly sequence reduction	fraction	error	fraction	error	fraction	error
**Whole Drosophila melanogaster genome - `minimap2` (n = 25 764)**						
HPC	0.957 +0%	2.27e-03 + 0%	**0.963 +0%**	2.34e-03 + 0%	**0.998 +0%**	1.48e-02 + 0%
raw	**0.958 +0%**	2.27e-03 − 0%	0.962 −0%	2.34e-03 + 0%	0.997 −0%	**1.17e-02 −21%**
MSR$_F$	0.952 −1%	1.18e-03 − 48%	0.960 −0%	1.37e-03 − 41%	**0.998 +0%**	1.36e-02 − 8%
MSR$_E$	0.946 −1%	**0 −100%**	0.954 −1%	**0 −100%**	**0.998 +0%**	1.53e-02 + 3%
MSR$_P$	0.950 −1%	4.90e-04 − 78%	0.957 −1%	8.11e-04 − 65%	**0.998 −0%**	1.39e-02 − 6%
**Whole Drosophila melanogaster genome - `winnowmap2` (n = 25 764)**						
HPC	0.923 +0%	1.51e-03 + 0%	0.930 +0%	1.59e-03 + 0%	0.989 +0%	1.50e-02 + 0%
raw	**0.949 +3%**	1.92e-03 +27%	**0.954 +3%**	1.99e-03 +26%	**0.995 +1%**	**1.33e-02 −12%**
MSR$_F$	0.918 −1%	**1.27e-03 −16%**	0.925 −0%	**1.30e-03 −18%**	0.987 −0%	1.37e-02 − 9%
MSR$_P$	0.905 −2%	1.33e-03 −12%	0.912 −2%	1.53e-03 −3%	0.983 −1%	1.40e-02 − 7%
MSR$_E$	0.905 −2%	1.42e-03 − 6%	0.912 −2%	1.49e-03 − 6%	0.983 −1%	1.44e-02 − 4%
**Synthetic centromeric sequence - `minimap2` (n = 12 673)**						
HPC	0.870 +0%	**1.36e-03 + 0%**	0.964 +0%	**1.56e-03 + 0%**	**1.000 +0%**	9.00e-03 + 0%
raw	**0.936 +8%**	1.86e-03 + 36%	**0.984 +2%**	2.09e-03 + 34%	**1.000 +0%**	**4.50e-03 −50%**
MSR$_E$	0.885 +2%	3.39e-03 +149%	0.962 −0%	3.53e-03 +127%	**1.000 +0%**	1.20e-02 +33%
MSR$_F$	0.850 −2%	2.04e-03 + 50%	0.968 +0%	2.12e-03 + 36%	**1.000 +0%**	6.63e-03 −26%
MSR$_P$	0.898 +3%	1.58e-03 + 16%	0.968 +0%	1.79e-03 + 15%	**1.000 +0%**	9.78e-03 + 9%
**Synthetic centromeric sequence - `winnowmap2` (n = 12 673)**						
HPC	0.775 + 0%	**1.32e-03 + 0%**	**0.822 +0%**	**1.82e-03 + 0%**	0.997 +0%	8.37e-02 + 0%
raw	**0.850 +10%**	2.04e-03 +54%	0.890 +8%	1.95e-03 + 7%	**0.999 +0%**	**4.60e-02 −45%**
MSR$_E$	0.795 + 2%	2.28e-03 +73%	0.846 +3%	2.52e-03 +38%	0.997 −0%	6.96e-02 −17%
MSR$_F$	0.820 + 6%	1.83e-03 +38%	0.867 +6%	2.27e-03 +25%	0.997 −0%	5.97e-02 −29%
MSR$_P$	0.780 + 1%	1.62e-03 +22%	0.829 +1%	2.09e-03 +15%	0.997 −0%	8.65e-02 + 3%
**Whole human genome - `minimap2` (n = 655 594)**						
HPC	**0.935 +0%**	1.85e-03 + 0%	**0.942 +0%**	1.85e-03 + 0%	**1.000 +0%**	1.46e-02 + 0%
raw	0.921 −1%	1.86e-03 + 0%	0.927 −2%	1.86e-03 + 1%	0.998 −0%	**1.29e-02 −11%**
MSR$_E$	0.926 −1%	**6.92e-05 −96%**	0.936 −1%	**1.17e-04 −94%**	0.999 −0%	1.76e-02 +20%
MSR$_P$	0.929 −1%	2.20e-04 −88%	0.938 −0%	4.15e-04 −78%	0.999 −0%	1.55e-02 + 6%
MSR$_F$	0.930 −1%	1.09e-03 −41%	0.938 −0%	1.29e-03 −30%	**1.000 −0%**	1.51e-02 + 4%
**Whole human genome - `winnowmap2` (n = 655 594)**						
HPC	0.894 + 0%	1.43e-03 + 0%	0.902 +0%	1.49e-03 + 0%	0.988 +0%	1.92e-02 + 0%
raw	**0.932 + 4%**	1.75e-03 +23%	**0.937 +4%**	1.79e-03 +20%	**0.994 +1%**	**1.43e-02 −26%**
MSR$_F$	0.874 − 2%	2.81e-04 −80%	0.886 −2%	3.82e-04 −74%	0.984 −0%	1.94e-02 + 1%
MSR$_E$	0.795 −11%	**6.33e-05 −96%**	0.820 −9%	**8.93e-05 −94%**	0.971 −2%	2.08e-02 + 9%
MSR$_P$	0.826 − 8%	8.68e-05 −94%	0.845 −6%	1.14e-04 −92%	0.975 −1%	2.11e-02 +10%
**Whole Human genome (repeated regions) - `minimap2` (n = 68 811)**						
HPC	**0.619 + 0%**	3.29e-04 + 0%	0.656 + 0%	3.10e-04 + 0%	**0.998 +0%**	7.79e-02 + 0%
raw	0.514 -17%	1.98e-04 -40%	0.539 -18%	2.16e-04 -30%	0.981 -2%	**6.69e-02 -14%**
MSR$_F$	0.601 - 3%	2.18e-04 -34%	0.640 - 2%	2.27e-04 -27%	**0.998 -0%**	8.15e-02 + 5%
MSR$_E$	0.618 - 0%	1.41e-04 -57%	**0.658 + 0%**	**1.55e-04 -50%**	0.997 -0%	8.23e-02 + 6%
MSR$_P$	0.616 - 1%	**1.18e-04 -64%**	0.656 + 0%	1.99e-04 -36%	0.997 -0%	8.31e-02 + 7%
**Whole Human genome (repeated regions) - `winnowmap2` (n = 68 811)**						
HPC	0.525 + 0%	1.24e-03 + 0%	0.557 + 0%	1.49e-03 + 0%	0.950 +0%	1.19e-01 + 0%
raw	**0.648 +23%**	1.26e-03 + 1%	**0.672 +21%**	1.49e-03 + 0%	**0.968 +2%**	**8.09e-02 -32%**
MSR$_F$	0.482 - 8%	**1.63e-03 +31%**	0.516 - 7%	1.83e-03 +23%	0.940 -1%	1.21e-01 + 2%
MSR$_E$	0.366 -30%	6.35e-04 -49%	0.405 -27%	**9.32e-04 -37%**	0.911 -4%	1.38e-01 +17%
MSR$_P$	0.415 -21%	9.45e-04 -24%	0.451 -19%	1.16e-03 -22%	0.920 -3%	1.39e-01 +17%

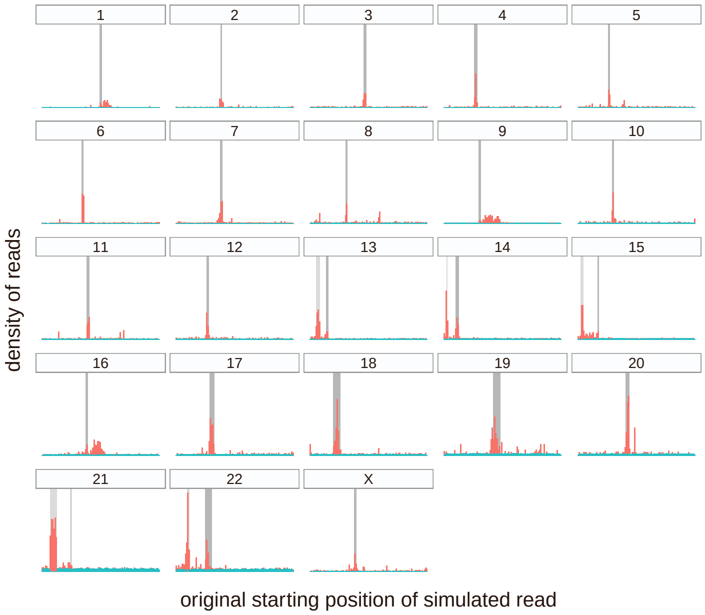

Figure S1. Origin of correctly and incorrectly mapped raw reads, related to Figure 6

Distribution of the origin of correctly and incorrectly mapped simulated reads (in teal and red respectively) on the different chromosomes of the whole human genome. The dark grey rectangle for each chromosome represents the centromere of that chromosome. The lighter gray rectangle on chromosomes 13, 14, 15, 21 and 22 correspond to satellites denoted as “stalk”, another repetitive region.

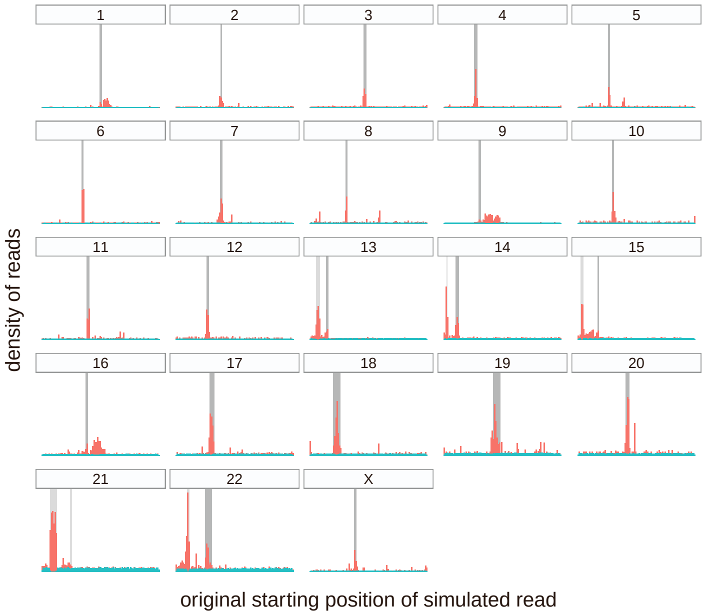

Figure S2. Origin of correctly (teal) and incorrectly (red) mapped reads, transformed with HPC, related to Figure 6
Distribution of the origin of correctly and incorrectly mapped simulated reads (in teal and red respectively) on the different chromosomes of the whole human genome. The dark grey rectangle for each chromosome represents the centromere of that chromosome. The lighter gray rectangle on chromosomes 13, 14, 15, 21 and 22 correspond to satellites denoted as “stalk”, another repetitive region.

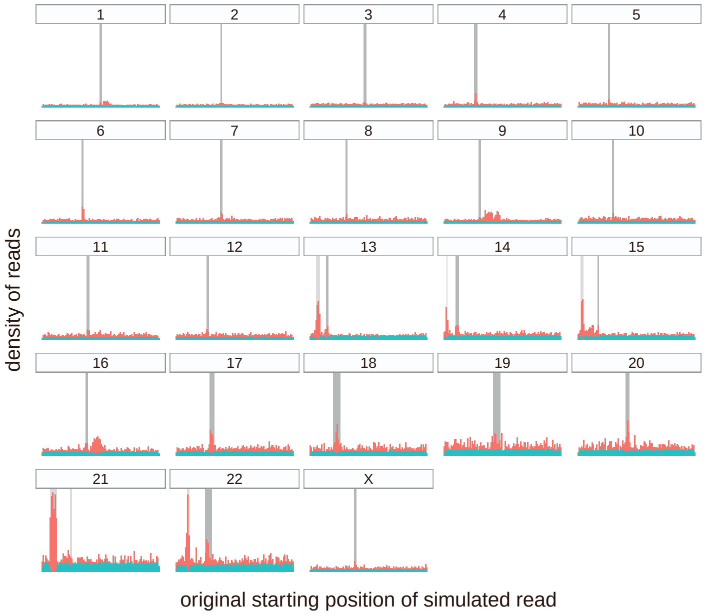

Figure S3. Origin of correctly (teal) and incorrectly (red) mapped reads, transformed with MSR_E, related to Figure 6

Distribution of the origin of correctly and incorrectly mapped simulated reads (in teal and red respectively) on the different chromosomes of the whole human genome. The dark grey rectangle for each chromosome represents the centromere of that chromosome. The lighter gray rectangle on chromosomes 13, 14, 15, 21 and 22 correspond to satellites denoted as “stalk”, another repetitive region.

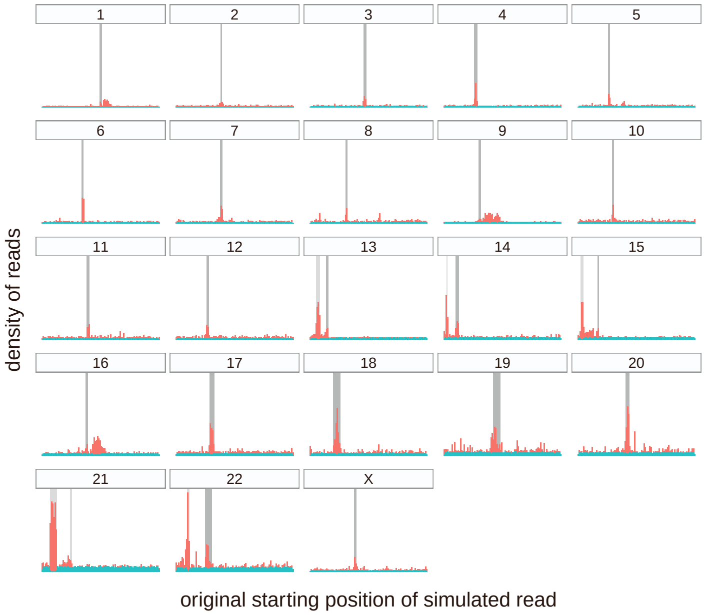

Figure S4. Origin of correctly (teal) and incorrectly (red) mapped reads, transformed with MSR$_P$, related to Figure 6
Distribution of the origin of correctly and incorrectly mapped simulated reads (in teal and red respectively) on the different chromosomes of the whole human genome. The dark grey rectangle for each chromosome represents the centromere of that chromosome. The lighter gray rectangle on chromosomes 13, 14, 15, 21 and 22 correspond to satellites denoted as “stalk”, another repetitive region.

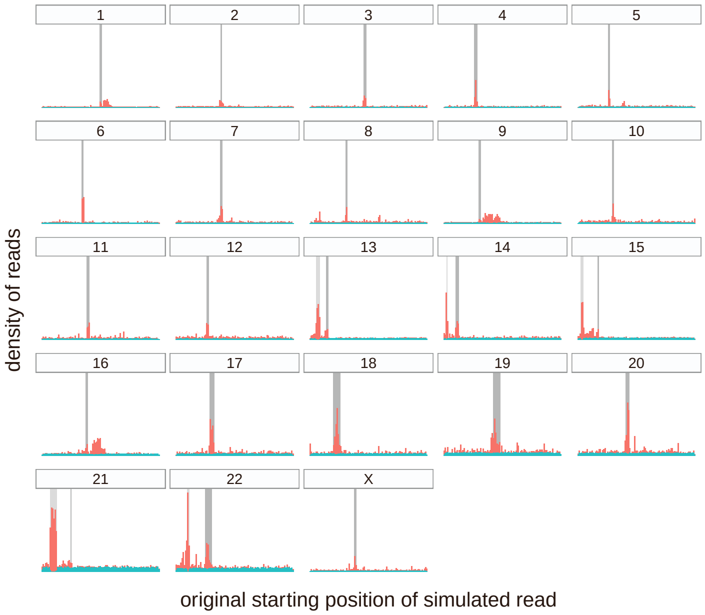

Figure S5. Origin of correctly (teal) and incorrectly (red) mapped reads, transformed with MSR_F, related to Figure 6

Distribution of the origin of correctly and incorrectly mapped simulated reads (in teal and red respectively) on the different chromosomes of the whole human genome. The dark grey rectangle for each chromosome represents the centromere of that chromosome. The lighter gray rectangle on chromosomes 13, 14, 15, 21 and 22 correspond to satellites denoted as “stalk”, another repetitive region.

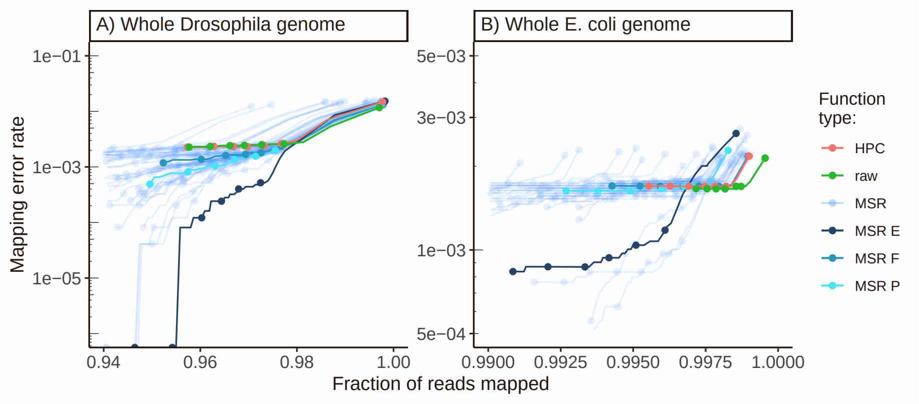

Figure S6. Performance of MSRs on whole Drosophila and E. coli genomes, related to Figure 5

Results of the `paftools mapeval` evaluation on reads simulated and mapped to whole *Drosophila melanogaster* and *Escherichia coli* (Genbank ID U00096.2) genomes. MSRs E, F and P are shown in different shades of blue to differentiate them from other MSRs. Reads were simulated with `nanosim`, and mapped with `minimap2`.
